# Evidence-based management of stage 2 pressure injuries in country-specific context

**DOI:** 10.3389/fmed.2025.1650052

**Published:** 2026-01-12

**Authors:** Liqun Luo, Liang Hao, Xiulin Wen, Le Tang, Xueyan Liu

**Affiliations:** 1 Department of Nursing, Xi'an People's Hospital (Xi'an Fourth Hospital), Xi'an, China; 2 Department of Gynecology, Xi'an People's Hospital (Xi'an Fourth Hospital), Xi'an, China; 3 Department of Nursing, The First Affiliated Hospital of Xi’an Jiaotong University, Xi'an, China

**Keywords:** evidence-based practice, knowledge translation and dissemination, non-wound specialist nurse, pressure injuries, wound management

## Abstract

**Background:**

The prevalence of pressure injuries (PI) among hospitalized adults worldwide remains high, posing a serious challenge to global healthcare systems. In China, the scarcity of specialized wound care nurses often leads to the suboptimal management of stage 2 PI by general nursing staff. Therefore, enhancing the wound management capabilities of these non-specialist nurses is crucial for improving healing rates and patient outcomes. This study aimed to systematically summarize the best evidence for stage 2 PI wound management and develop a standardized, evidence-based practice protocol for clinical nurses.

**Method:**

We employed an evidence-based continuous quality improvement model, which comprised four phases: evidence gathering, baseline review, evidence introduction, and effectiveness evaluation. The study was conducted in a tertiary hospital in China, involving 80 skin liaison nurses and 70 patients with stage 2 PI.

**Results:**

Following the implementation of the evidence-based strategy, nurses’ PI knowledge scores (PZ-PUKT) significantly increased from 53.31 ± 4.75 to 56.29 ± 4.72 (*p* < 0.01). The implementation rate of key review indicators improved markedly, with some increasing from 0 to 100%. Patient outcomes also improved significantly, as evidenced by reduced PUSH scores (*p* < 0.05), lower pain scores (VAS), and higher wound healing rates in the intervention group.

**Discussion:**

Research findings indicate that structured, evidence-based nursing strategies significantly enhance nurses’ understanding and implementation of best practices, thereby accelerating wound healing and alleviating patient pain. This study provides a feasible model for implementing standardized stage 2 PI care in resource-limited healthcare settings and lays the groundwork for future multicenter research on intelligent nursing interventions.

## Introduction

Pressure injury (PI) is a limited injury to the skin or subcutaneous tissue that occurs under pressure or a combination of pressures, most often in the bony alveolar region, but can also be caused by instruments or other objects (1). A recent systematic review highlighted the substantial prevalence of this condition, reporting an overall prevalence of PI of 60.9% prior to interventions, with hospital-acquired PI accounting for 52.9% of cases. Following the implementation of multifaceted interventions, these rates were significantly reduced to 28.7 and 21.3% (2). In the United States specifically, the economic burden associated with hospital-acquired PI was estimated at $26.8 billion, underscoring the substantial financial impact on healthcare systems (3, 4). Among them, a systematic analysis in Europe found that patients need to spend an average of 2.65 to 87.57 euros per day for the prevention of PI (5). Related data suggest that the incidence rate of in-hospital PI in Chinese tertiary care hospitals is 0.03%; Guo et al. (6) found that the prevalence rate of in-hospital PI is 1.67%, and the incidence rate is 0.68% through meta-analysis, and there is a trend of increasing year by year (7). There is a trend of increasing year by year. An RCT from 480 neurosurgical patients found that the occurrence of PI, without timely and effective intervention, can lead to a range of problems over time, including rhabdomyolysis, chronic osteomyelitis, sinusitis, joint infections, sepsis, and fistulae (8). In particular, patients in orthopaedics and intensive care units are more prone to muscle atrophy, weakened local blood supply and circulation, decreased skin elasticity, poor nutritional status, and decreased sensory function due to prolonged hospitalization, more bedridden time, and poor self-care ability, and more serious complications may also occur if stress injuries occur without timely nursing management measures (9, 10). Therefore, actively preventing the occurrence of PI and managing wounds that have occurred, promoting healing, and reducing the waste of medical resources have become key issues in clinical care.

According to the staging system established by the National PI Advisory Panel (NPIAP), PI are classified into four primary stages: Stages 1 through 4, along with unstaged pressure injuries and deep tissue injury (11). Statistically, the incidence of stage 1 and 2 PI remains at its highest, with the sacrococcygeal region being the preferred site (11). Among these, wound management for stage 2 PI is critical to treatment. If detected promptly and treated effectively, healing typically occurs within 9–15 days (12). However, delayed treatment or improper management can easily lead to chronic, difficult-to-heal wounds, increasing the risk of complications such as infection, sepsis, and systemic failure, thereby significantly raising patient mortality rates (13). Under current U.S. healthcare policies and quality standards, stage 3 and stage 4 PI are explicitly classified as serious “hospital-acquired” adverse events, whose occurrence is considered fundamentally preventable and unacceptable. Furthermore, if a stage 2 PI present at admission worsens during hospitalization, the hospital bears corresponding clinical management and financial responsibility (14). Therefore, once diagnosed, stage 2 PI should be actively targeted based on the cause and aggravating factors. In the treatment of clinical PI wounds, in addition to administering necessary medications and physical therapy measures, mild PI are treated through wound localisation (dressing change), which relieves patient pain, reduces infection, and promotes wound healing (15). How to effectively deal with PI wounds is a hotspot in the direction of wound management research. Currently, unequal care outcomes and a lack of PI care expertise are common in clinical PI care, negatively affecting the quality and effectiveness of care (16, 17). To address the practical challenges in pressure ulcer care, multiple countries and regions have developed corresponding clinical practice guidelines. For example, Australia has published the Australian Wound Prevention and Management Standards (4th Edition), while the National Pressure Injury Advisory Panel (NPIAP) in the United States, the European Pressure Ulcer Advisory Panel (EPUAP), and the Pan-Pacific Pressure Injury Alliance (PPPIA) have jointly released the international Clinical Practice Guideline for the Prevention and Treatment of Pressure Ulcers/Pressure Injuries (1, 18). These guidelines aim to systematically update nursing knowledge, standardize operational procedures, and promote wound healing. Furthermore, systematic capacity building is regarded as a core strategy for optimizing the prevention and monitoring of hospital-acquired pressure ulcers. Its framework encompasses multiple dimensions, including research, organizational development, professional training, leadership enhancement, multidisciplinary collaboration, and patient involvement, aiming to comprehensively elevate healthcare professionals’ expertise and practical capabilities (19). However, multiple international studies indicate that nurses’ knowledge of pressure ulcer prevention and management remains inadequate. This underscores the importance of implementing continuing professional development to bridge the gap between best evidence and clinical practice (20, 21). This global challenge is exacerbated in China by a severe shortage of specialized wound care nurses, creating a structural weakness that contributes to persistently high PI incidence rates (22). Furthermore, since most evidence for stage 2 PI management originates from foreign contexts, the direct application of international guidelines faces numerous obstacles due to differences in healthcare financing models, accessibility, and costs of dressings, and nursing education systems between China and other countries. Therefore, localizing international evidence is essential to ensure its practicality and effectiveness within China’s clinical settings. Evidence-based practice (EBP) is a clinical decision-making process that integrates best evidence, clinical expertise, and patient values (23). Given that stage 2 PI care in China is primarily delivered by non-specialist nurses, this study aims to develop and evaluate an evidence-based management protocol led by non-wound specialist nurses. Research objectives include: (1) formulating action strategies based on existing best evidence; (2) standardizing clinical management of stage 2 PI; (3) enhancing nurses’ wound management capabilities; and (4) accelerating patient wound healing. This study was reviewed by the Center for Evidence-Based Nursing at Fudan University (project registration number ES20221178).

## Methods/design

### Design

**Figure 1:**
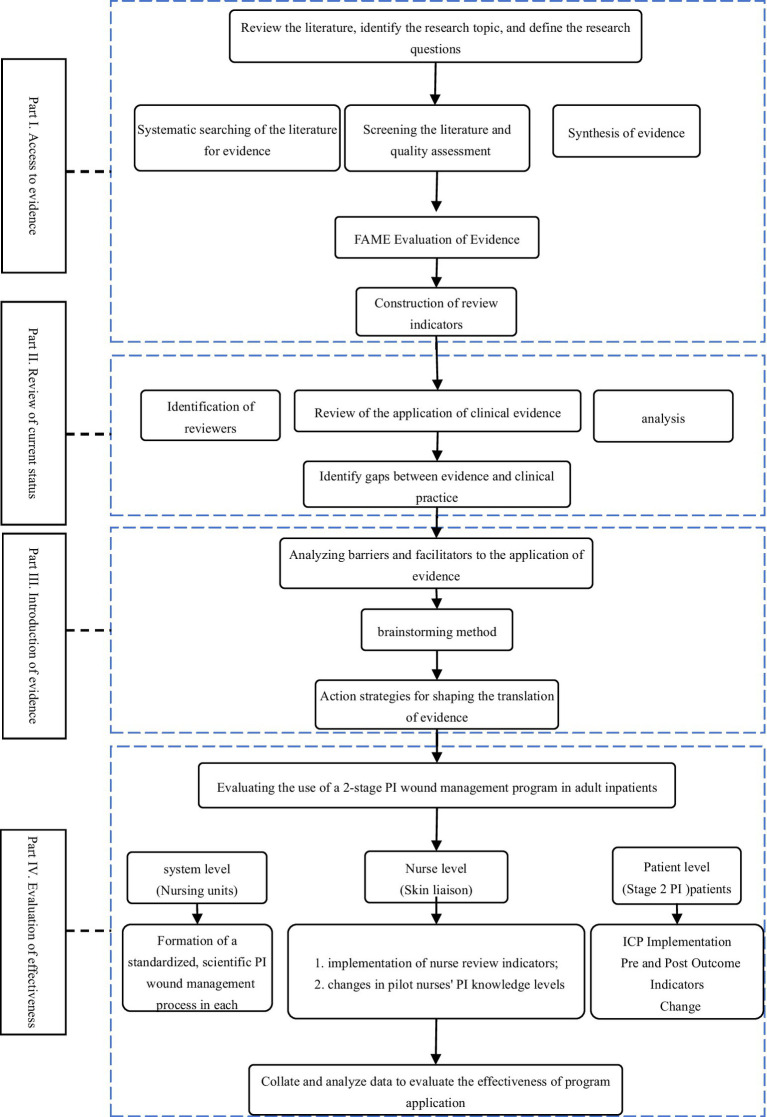
Flow chart of the study design.

Our goal is to use scientific research methods to improve action strategies for patients with stage 2 PI. This study is a quasi-experimental study that follows the four steps of the evidence-based continuous quality improvement model research paradigm (Figure 1). This four-stage model provides a structured theoretical framework for evidence-based practice, guiding healthcare professionals in the practical application of research evidence in clinical practice.

### Study timeline

This study was conducted between December 2022 and August 2023 and comprised the following four phases:

(1) Phase I (evidence gathering): December 2022–January 2023.
(2) Phase II (baseline review): February–April 2023.
(3) Phase III (evidence introduction): May 2023.
(4) Phase IV (effectiveness evaluation): June–August 2023.

Patient recruitment for the pre-evidence application group was conducted from February to April 2023, while recruitment for the post-evidence application group took place from June to August 2023.

### Setting

This study was conducted at a provincial hospital in Shaanxi Province, China, which is one of the largest tertiary hospitals in the province and boasts highly developed facilities, platforms, and staff support.

### Methods

This study follows the four steps of the evidence-based continuous quality improvement model research paradigm.

#### Phase I: gathering evidence

##### Establishment of an evidence-based practice working group

An evidence-based practice group was formed; project one supervisor (responsible for overall project guidance as well as practice guidance); two group members who received systematic training from the Evidence Translation and Clinical Utilization Workshop at the School of Nursing, Fudan University (responsible for study design as well as quality review), three nurse leaders and wound therapists (responsible for project coordination, follow-up, evaluation, and staff training); and one dermatologist (responsible for technical support and patient consultation); two graduate nursing students (responsible for literature search, data collection, and project follow-up).

##### Establishing evidence-based questions and obtaining evidence

Evidence-based questions were formulated using the PIPOST model from Fudan University’s Evidence-Based Nursing Center (24).

(1) P (population): The target population for evidence application in this study is adult hospitalized patients with stage 2 PI;
(2) I (intervention): The intervention implemented in this study is a comprehensive wound management protocol based on best evidence, encompassing wound assessment, pain management, and wound dressing techniques;
(3) P (professional): The practitioners responsible for applying the evidence in clinical practice are the hospital’s clinical skin liaisons;
(4) O (outcome): Evidence translation outcomes are multifaceted, specifically including: ① Systemic Outcomes: Revision of wound documentation forms, creation of a new wound dressing manual, and updates to the skin liaison training program; ② Practitioner Outcomes: Skin liaisons’ knowledge level regarding PI and compliance rates with review indicators before and after evidence implementation; ③ Patient Outcomes: Patient wound healing rates, healing quality, and pain levels;
(5) S (setting): The clinical application and translation setting is a Grade A tertiary hospital in Shaanxi Province, China;
(6) T (type of evidence): Evidence resources supporting this study include clinical guidelines, evidence summaries, systematic reviews, expert consensus statements, and relevant primary research.

A total of 8 articles were finally included after literature screening, including three guidelines (1, 25, 26), one systematic evaluation, (27) three evidence summaries (28–30), and 1 RCT (31). A total of 23 pieces of evidence were eventually extracted.

##### Formation of quality review indicators

The development of quality review indicators is a systematic process. First, we convened a multidisciplinary expert panel comprising two international stoma therapists, two nursing specialists, and two evidence-based nursing experts, as well as three PhDs and three Master’s degree holders, including two mid-level, two associate senior, and two senior-level professionals. Through evidence assessment meetings, the 23 initially extracted pieces of evidence underwent evaluations of feasibility, appropriateness, clinical significance, and effectiveness (FAME) (32). Ultimately, two pieces of evidence were excluded, leaving 21 pieces of optimal evidence suitable for clinical application. Subsequently, two international wound therapists independently drafted preliminary indicators to translate these 21 evidence items into specific, measurable clinical behaviors. The evidence-based practice team then convened a consensus meeting to review and revise each draft item. This process resulted in 31 review indicators, consolidated into the “Review Indicator Implementation Survey Form.” This form specifies the review subjects and methods for each indicator, as detailed in Table 1.

**Table 1:** Review of indicator content and methodology.

Evidence category	Evidence description	Review criteria	Review method
Wound assessment (1–3)	1. Wound assessment: Wound size, depth, color, tissue type, exudate volume, exudate characteristics, odor, presence of infection signs.	1. After opening the dressing and cleaning the wound at each dressing change, assess the color of the surrounding skin. 2. After opening the dressing and cleaning the wound during each dressing change, assess the temperature of the surrounding skin. 3. After opening the dressing and cleaning the wound during each dressing change, assess the integrity of the surrounding skin (normal, edema, erosion, hyperpigmentation, erythema, maceration, allergic reaction, altered elasticity, etc.).	On-site inspection
	2. Certain wound assessment tools are suited for describing wound status (observational tools, e.g., NPUAP PI staging system), while others are better for evaluating wound healing (evaluative tools, e.g., PUSH scale). Therefore, select wound assessment tools based on the intended purpose.	4. Does the department where the is located have standardized wound measurement tools (e.g., sterile cotton swabs, probes, centimeter rulers, concentric circle rulers, sterile transparent films, measurement grids included with new dressings, photographic documentation, etc.)? 5. Does the department utilize standardized wound assessment tools (e.g., PUSH scale for evaluating wound size, exudate volume, and wound tissue type; DESIGN tool for assessing pressure ulcer severity and monitoring healing progression; sessing scale for evaluating wound healing over time)?	Review system documentation at/conduct on-site inspection
	3. Minimize the impact of surrounding soft tissue deformation on measurements. If tissue deformation is unavoidable, position the patient identically during each assessment.	6. Position the patient in the same posture for each assessment.	On-site viewing
Pain management (4–7)	4. Assess PI pain during the initial examination and continue monitoring pain during subsequent follow-ups, including before and after each wound care intervention.	7. Conduct a pain assessment with every wound dressing change.	Review wound documentation
	5. A comprehensive pain assessment should include the following: ① Characteristics, intensity, and duration of PI pain; ② Changes in severity or nature of PI pain over time; ③ Neurological physical examination; ④ Appropriate diagnostic methods to determine the type and cause of pain; ⑤ Severity and duration of PI; ⑥ Psychosocial assessment; ⑦ Activities related to PI pain; ⑧ Activities related to alleviating PI pain.	8. Assess the characteristics, intensity, and duration of PI pain. 9. Assess changes in the severity or nature of PI pain over time. 10. Select appropriate diagnostic methods to determine the type and cause of pain during the assessment of PI pain. 11. Conduct a psychosocial assessment during PI pain evaluation. 12. Assess activities related to PI pain during the evaluation.	
Pain management (4–7)	6. Implement a person-centered pain management plan using interventions such as: ① Position changes; ② Distraction and conversation; ③ Touch therapy; ④ Music therapy; ⑤ Heat application; ⑥ Progressive muscle relaxation; ⑦ Meditation and self-hypnosis; ⑧ Guided imagery.	13. Implement appropriate interventions based on pain assessment findings.	On-site inspection/patient inquiry
	7. Apply moist wound healing principles to reduce PI pain: Consider highly absorbent dressings requiring less frequent changes, including but not limited to alginate dressings, hydrogel dressings, polymer foam dressings, and soft silicone-edged wound dressings. Non-adherent and/or moist dressings cause less pain and trauma during removal; standard gauze dressings are more likely to cause pain and require frequent changes to maintain wound bed moisture.	14. Employ pain-free dressing removal techniques at every dressing change. 15. Use standard gauze dressings with frequent changes (maintaining constant wound bed moisture).	
Peripheral and tissue cleansing (8–10)	8. Perform skin cleansing daily as needed using a mildly acidic or neutral skin cleanser.	16. Have the patient clean the surrounding skin daily with a mildly acidic or neutral cleanser.	Inquire with the patient
	9. PI may be cleaned using methods such as irrigation, sponge bathing, showering, or whirlpool therapy. Apply appropriate pressure during cleaning to remove foreign bodies and tissue debris while taking care not to damage the wound.	17. When encountering extensive wound surface adhesions that are difficult to remove, would you opt for rinsing or scrubbing to clean the wound? Perform these actions gently to avoid wound damage.	On-site observation
	10. Clean the perilesional skin with an appropriate pH-neutral skin cleanser to achieve optimal wound and wound pH.	18. For wounds with significant exudate or obvious contamination, use a surfactant-containing wound cleanser (e.g., benzalkonium bromide, PHMB) for washing or irrigation.	
Wound management (11–17)	11. For all PI, select an appropriate wound dressing based on the patient’s and/or primary caregiver’s goals and self-care capabilities, as well as clinical assessment, including: the diameter, shape, and depth of the PI; the need to address microbial load; the ability to maintain wound bed moisture; the nature and volume of wound exudate; the condition of the wound bed tissue; the condition of the surrounding skin; and the presence of sinuses and/or subcutaneous pain.	19. Select appropriate dressings based on patient and primary caregiver capabilities and the characteristics of the PI.	
	12. Evaluate the cost-effectiveness of wound dressings in the local setting, considering both direct and indirect costs to the healthcare system and to patients with PI.	20. Consider cost-effectiveness, insurance systems, and patient out-of-pocket expenses when selecting dressings.	
Wound management (11–17)	13. Apply hydrocolloids, hydrogels, and polymer dressings to uninfected Stage II PI based on clinical condition. 14. For moderately to highly exuding stage 2 PI, foam dressings or alginate dressings may be used to manage exudate. 15. For wound infection, locally applied dressings should include silver-impregnated hydrofibers (1A), silver-impregnated polyurethane foam (1A), and silver alginate dressings (1A).	21. Use hydrocolloid dressings for wounds with little to no exudate. 22. Use hydrocolloid dressings for dry or eschar-covered wounds. 23. For wounds with significant exudate exceeding the wound bed and threatening to macerate surrounding skin, use foam dressings or combine with alginate dressings. 24. For infected wounds, select silver dressings.	
	16. Consider applying collagen dressings to improve the healing rate of non-healing PI and reduce signs and symptoms of wound infection.	25. Apply collagen dressings to improve the healing rate of non-healing PI and reduce wound infection rates.	
	17. Select dressings that absorb and control exudate without leakage or penetration, maintaining a moist but not saturated wound environment. Immediately replace dressings if they become damp, damaged, displaced, loose, or excessively saturated.	26. After each wound dressing change, ensure the dressing appears clean and is securely in place.	
Wound documentation (18, 19)	18. Document even minor skin changes promptly and accurately. Record wound status after each dressing change. It is recommended to photograph the wound at least weekly to dynamically assess the effectiveness of the treatment plan.	27. Changes in the patient’s skin condition are documented promptly and accurately during each dressing change. 28. Photographs of the wound are taken before and after each dressing change.	Review wound record form
	19. Document wound measurement results for ongoing comparison and assessment of healing progress. Employ valid and reliable tools to monitor changes in PI size: Use the PUSH scale to evaluate patient wound healing. For adult patients, use the Braden scale to assess PI risk.	29. Use the PUSH scale and Braden scale to monitor PI healing and risk status.	Review the wound documentation form
Quality management (20, 21)	20. Incorporate evidence-based policies, procedures, and protocols, along with standardized documentation systems, as components of quality improvement initiatives to reduce PI incidence.	30. Incorporate standardized documentation/protocols/evidence-based policies as components of quality improvement plans for PI incidence.	Review system documentation
	21. Consider implementing commendations and rewards to encourage healthcare professionals to actively participate in the organization’s quality improvement initiatives.	31. Implement incentive or penalty systems to motivate or penalize clinical nurses for active participation in quality improvement initiatives.	Review system documentation

#### Phase II: review of current situation

##### Subject of review

A tertiary general hospital in Shaanxi Province was selected as the site for evidence application. A non-probability sampling method was used to select all skin liaison officers in the hospital as the study population. This study adopted a quasi-experimental, pre-post design. The pre-evidence application (control) group included patients admitted from February 2023 to April 2023, while the post-evidence application (intervention) group included patients admitted from June 2023 to August 2023. Patients with stage 2 PI were selected as study subjects using a convenience sampling method. The specific inclusion and exclusion criteria are shown in Table 2.

**Table 2:** Inclusion and exclusion criteria.

Research subjects	Inclusion criteria	Exclusion criteria
Patients	1. Diagnosed with Stage 2 pressure injury (PI) according to the latest 2019 staging criteria of the National Pressure Ulcer Advisory Panel (NPUAP); (1)2. Age ≥ 18 years; 3. Informed consent and voluntary participation in this study.	1. Patients with medical device-associated pressure injuries; 2. Patients with skin-related conditions that impair judgment or observation; 3. Currently using immunosuppressive agents; 4. Participants currently enrolled in other similar studies.
Nurse (skin liaison)	1. Registered nurse licensed to practice at this hospital; 2. Serving as the hospital’s skin liaison; 3. Voluntarily participating in this study.	1. Individuals who are on rotation, pursuing further training, or absent due to marriage, maternity leave, or sick leave during the study period; 2. International Certified Ostomy Therapists, International Certified Wound Therapists, or Wound Specialty Nurses.

This study was a class pilot study with a PUSH score as the primary outcome indicator, with a smaller total score indicating better healing. Checking the literature, it is known that the standard deviation of the PUSH score of the experimental group and the control group is 1.98 and 1.65, and the combined standard deviation is calculated to be 1.83 (33). The bounding value was determined by the statistical expert and the clinical expert to be 1.42 under the assumption of μt = μc when *α* = 0.05. according to the calculation formula:


$$\frac{{\left(u_{1-\alpha }+u_{1-\beta }\right)}^{2}s^{2}(1+1/k)}{{\left[\mu_{t}-\mu_{c}-(-\delta )\right]}^{2}}$$

It was calculated that the sample size required for each group was 29 cases. Considering a 20% sample size attrition rate, 35 study subjects were required for each group. Consequently, 35 patients were included in the baseline review and the post-evidence application review, respectively. A total of 80 nurses (skin liaison officers) were included in this study. The study was approved by the hospital ethics committee (No. XJTUIAF2023LSK-305).

##### Review tools

Based on the 31 review indicators, the “Review Indicator Implementation Questionnaire” was developed. The review method and review results were clarified, with the review results expressed as “implemented” or “not implemented.” The implementation rate of each review indicator is calculated as the number of times that meet the review indicators divided by the total number of review times, expressed as a percentage of 100%.

##### Review results

**Figure 2:**
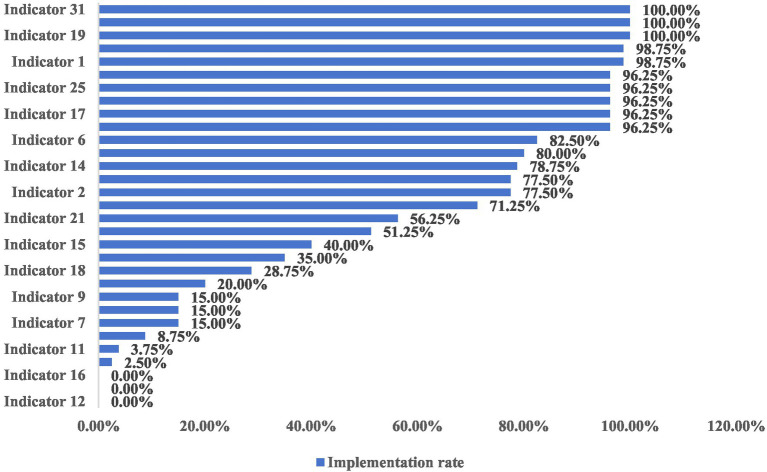
Results of the review of the current status of adherence to the review indicators.

According to the review indicators, 80 skin liaison officers, 80 nursing units (departments), and 35 patients were reviewed for the quality of PI wound management. The results showed that of the 31 review indicators, the implementation rate was 0 in 3 entries, with the following indicators: 12, 13, and 16; the implementation rate was <30% in 8 entries, with the following indicators: 7, 8, 9, 10, 11, 18, 22, and 26; the implementation rate was 30 to 60% in 8 entries; and the implementation rate was 30 to 60% in 3 entries. 30–60% of entries were 4. The results suggest a significant gap between best evidence and clinical practice in wound assessment, pain management, wound management, and sterilisation techniques, as illustrated in Figure 2.

#### Phase III: introduction of evidence

##### Facilitation and barrier analysis

Based on the review results and combined with the barrier identification evaluation form, the research team discussed to analyze the barriers and facilitators from the aspect of the department’s existing resources, corresponding countermeasures, and based on the i-PARIHS framework, through the I (innovation): change, R (recipients): recipients, and C (content): the organizational environment, and so on (34). Through team discussion, it was decided that the following barriers existed: (1) the skin liaison staff did not pay enough attention to PI wound assessment and pain management; (2) there was a lack of a unified wound assessment process and specific wound assessment tools; (3) there were a lot of new dressing products on the market, which required professionals to control the effectiveness of the products, the principles of the products, and the price; and (4) there was no unified pain assessment process in the hospital.

#### Practice change

##### Strengthen training

(1) The evidence-based practice group writes and produces a wound dressing manual, which can be accessed by healthcare workers through the QR code so that they can read it anytime and anywhere and compare the advantages and disadvantages of the more common dressings on the market.
(2) Record videos of PI disposal, organize a combination of centralized learning and self-study for nursing staff and recruit clinical nurses from all departments in the hospital to participate in wound treatment room rotations for targeted apprenticeships in wound assessment, cleansing, selection of dressings, and recording of wounds, among other operations.
(3) Hospital-wide training on knowledge and skills related to PI prevention and management for skin liaison officers, forming a faculty team led by wound specialist nurses to carry out diversified training for non-wound specialist nurses, e.g., a combination of case studies, workshops, etc., to update their knowledge in time, and this training was conducted four times for 40 min each time. In the morning, before shift handover, the nurse manager will randomly select part of the training content and correct and supplement any inaccurate or incomplete content to ensure that every nurse is proficient.

##### System optimization

The PI assessment system currently used in our hospital is cumbersome and complicated to use. Through the technological support of the hospital’s information department, the assessment system was optimised to reduce the workload of medical and nursing staff, making the patient’s PI assessment information clear at a glance.

##### Improvement of PI wound management record form

The team members will optimize the wound record sheet by checking the box “√” under each tip that needs to be operated to prevent nursing staff from omitting or reversing the operation points and, at the same time, to facilitate the real-time query and use by clinical nurses.

##### Developing a PI wound management process

Based on the best available evidence, combined with the opinions of the panel experts and the results of the review of the status quo, the intervention process for the management of pressure wounds in adult inpatients with stage 2 injuries was developed by taking the barriers as the entry point and crystallizing the implementation details of the evidence. Healthcare staff assessed the patient’s skin status within 1 h of admission and determined the stage of PI was present; if it was stage 1/2, the skin liaison in each unit led the general nurses to perform wound management based on the process of assessment, cleansing, treatment, and documentation, and if the patient suffered from stage 3 or more PIs, the healthcare staff, based on decompression and skin protection, promptly requested the hospital wound therapist to Consultation. During the patient’s hospitalization, healthcare staff should guide the patient and their family on measures related to skin decompression and protection, such as daily cleaning of the skin around the wound as needed, application of emollient lotion, and other necessary measures.

##### Ensuring intervention fidelity

To ensure consistent application of this evidence-based strategy across all nursing units, we adopted a comprehensive approach to maintain intervention fidelity. First, all training was delivered centrally by a dedicated team of experts using standardized materials. Second, a unified, step-by-step wound management protocol was developed and implemented. Third, the core project team conducted regular on-site spot checks and on-the-job coaching to observe compliance and promptly correct deviations in practice. Finally, a dedicated online communication platform was maintained for ongoing support and the timely resolution of issues, ensuring all practitioners received consistent guidance.

#### Phase IV: effectiveness evaluation

##### Study population

A tertiary general hospital in Shaanxi Province was selected as the evidence application site. The non-probability sampling method was used to select all skin liaison officers in the hospital as the study population. Patients suffering from stage 2 PI from June 2022 to August 2023 were selected as study subjects using a convenience sampling method. Their inclusion–exclusion criteria were the same as in phase II. There were 35 patients and 80 nurses (skin liaison staff).

##### Nurses’ evaluation indicators

(1) General information questionnaire: gender, age, hospital level, departmental title, years of work experience, and whether or not the skin liaison nurses are specialized.
(2) Chinese version of the Pieper-Zulkowski Stress Injury Knowledge Questionnaire (PZ-PUKT): The questionnaire was translated and validated by Nie (35) for use in the Chinese context. The Chinese version of the PZ-PUKT has demonstrated good reliability and validity, with a total Cronbach’s *α* of 0.932 and dimension values ranging from 0.823 to 0.840, indicating high internal consistency and making it suitable for assessing nurses’ PI knowledge in this population. The Cronbach *α* values of the dimensions also showed good consistency, namely: risk factors/prevention 0.831, PI staging 0.823, and wound description 0.840, which were slightly higher than those of the original version of the questionnaire, indicating that the Chinese version of the questionnaire has a good reliability in measuring the nurses’ knowledge about PI (35). In the scoring system of PZ-PUKT, the response options of the questionnaire were set as “correct,” “wrong,” and “do not know.” One point is awarded for each correct answer, while no points are awarded for incorrect and do not know, out of a total of 72 points. Through this scoring mechanism, nurses’ knowledge of PI can be effectively assessed, which in turn guides education and training and improves nursing quality.
(3) Review of the indicator implementation questionnaire: consistent with the baseline review.

##### Patient evaluation indicators

(1) General information questionnaire: The questionnaire includes the patient’s gender, age, BMI, education level, smoking, source of PI, the presence of underlying diseases, wound location, Braden score and so on (36).
(2) Pressure Ulcer Healing Rating Scale (PUSH scale): This internationally recognised scale has been validated in multiple studies for assessing PI healing. The Chinese version of the PUSH scale has demonstrated excellent psychometric properties, with a content validity index of 0.965, Cronbach’s *α* coefficient of 0.823, and inter-rater reliability exceeding 0.85, confirming its suitability for evaluating treatment outcomes in Chinese patients with PI. The Cronbach’s *α* coefficient for this study was 0.784, indicating good reliability. It consists of three dimensions: wound area, exudate volume, and tissue type (37). Wound area is 0–24 cm^2^ with a score of 1–10; exudate volume is divided into four levels: none (0), small amount (1), medium amount (2), and large amount (3); and wound tissue type is divided into five levels: intact (0), epithelial (1), granulation (2), putrefied flesh (3), and necrotic tissue (4) (38). The sum of the scores for the three dimensions was used to evaluate the patient’s PI healing, with a total score ranging from 0 to 17, where a score of 0 indicated wound healing. The lower the total PUSH score, the less severe the symptoms, and vice versa.
(3) Visual Analogue Scale (VAS): As a globally recognised and widely used self-report tool for pain assessment, the VAS has been extensively validated across diverse patient populations and clinical settings, demonstrating high sensitivity in detecting changes in pain intensity. Its excellent reliability and validity are well-established in the literature. It was used for the subjective assessment of the patient’s pain level. The total score ranges from 0 to 10, where 0–3 indicates mild pain, 4–6 indicates moderate pain, and 7–10 indicates severe pain (39).
(4) Wound healing rate = (initial wound area − when treated wound area)/initial wound area × 100% (40).

### Data collection

At the time of the patient’s first wound treatment, a general information questionnaire was completed by the investigator patient/family member; during the three treatments of the first wound treatment, 7 days of wound treatment, and 14 days of wound treatment, PUSH scores and wound healing rates of patients in both groups were assessed and recorded by trained skin liaison officers with the researcher. Following each wound care procedure, the skin liaison officer and the investigator jointly assess and record the patient’s pain score using the Visual Analog Scale (VAS).

### Statistical analysis

The data from this study were entered and statistically analyzed in pairs using SPSS 26.0 software. General data were described as follows: Measures of normal distribution were described as mean and standard deviation; count data were described as frequency and percentage. Comparison of baseline data between the two groups: two independent samples *t*-test was used for the measurement data that conformed to normality, and non-parametric rank sum test was used for those that did not; chi-square test was used for the counting data; *p* < 0.05 indicated a statistical difference. Comparison of information at three time points: the changes in the PUSH score, VAS score and wound healing rate of the two groups were analyzed by Generalized Estimating Equation (GEE).

## Results

### Comparison of nurses’ stress injury knowledge scores before and after evidence application

Nurses’ PZ-PUKT questionnaire score before evidence application was (53.31 ± 4.75), and the standardized score was 74.04%; nurses’ PZ-PUKT questionnaire score after evidence application was (56.29 ± 4.72), and the standardized score was 78.18%. The total score increased significantly after evidence application with a large effect size (Cohen’s *d* = 0.64, 95% CI [0.25, 1.02]). Nurses’ PZ-PUKT scores and scores of dimensions after evidence application were significantly higher than those before evidence application (*p* < 0.01), as shown in Table 3.

**Table 3:** Comparison of PZ-PUKT total scores and dimensions before and after training (*N* = 80).

Item	Number of entries	Before evidence application x̄ ± s	After evidence application x̄ ± s	*t*	*p*
Total score	72	53.31 ± 4.75	56.29 ± 4.72	−3.974	<0.001
Risk factors/prevention	28	21.84 ± 2.07	22.84 ± 2.07	−3.054	0.003
Pressure ulcer stage	20	15.41 ± 2.12	16.41 ± 2.08	−3.012	0.003
Wound description	24	16.06 ± 2.34	17.04 ± 2.20	−2.783	0.006

Paired *t*-tests are shown.

### Comparison of review indicator implementation rate before and after evidence application

The entries with review indicator implementation rates <60% at the baseline review were review indicators 5, 7, 8, 9, 0, 11, 12, 13, 15, 16, 18, 21, 22, 26, and 28, and the focus of this reform was on the indicators with an implementation rate <60%, so the other indicators were excluded from the scope of this study. The implementation rate of all indicators increased after the application of evidence, and the implementation rate of the review indicators 12, 13, and 16 increased from 0 to 100%, as shown in Table 4.

**Table 4:** Comparison of review indicator implementation rates before and after evidence application (*N* = 80).

Review indicators	Pre-evidence application group			Post-evidence application group			*X^2^*	*p*
	Implemented (cases)	Not implemented (cases)	Implementation rate (%)	Implemented (cases)	Not implemented (case)	Implementation rate (%)		
Indicator 12	0	80	0.00	80	0	100.00	–	–
Indicator 13	0	80	0.00	80	0	100.00	–	–
Indicator 16	0	80	0.00	35	0	100.00	22.259	<0.001
Indicator 10	2	78	2.50	62	18	77.50	30.032	<0.001
Indicator 11	3	77	3.75	72	8	90.00	59.325	<0.001
Indicator 26	7	34	8.75	80	0	100.00	46.184	<0.001
Indicator 7	12	68	15.00	57	23	71.25	26.217	<0.001
Indicator 8	12	68	15.00	56	24	70.00	28.320	<0.001
Indicator 9	12	68	15.00	56	24	70.00	19.326	<0.001
Indicator 22	16	64	20.00	77	7	96.25	5.572	0.018
Indicator 18	7	32	28.75	61	19	76.25	27.200	<0.001
Indicator 5	23	57	35.00	80	0	100.00	15.180	<0.001
Indicator 15	32	48	40.00	80	0	100.00	19.552	<0.001
Indicator 28	41	39	51.25	80	0	100.00	–	–
Indicator 21	45	35	56.25	79	1	98.75	40.232	<0.001

### Comparison of the general information of the two study groups

A total of 80 skin liaisons were included in this study, using their own before and after control study. Inpatients admitted to our hospital with stage 2 PI from February 2023 to August 2023 had their evidence application compared before and after. General information of patients in the two groups was compared; *p* > 0.05, indicating no statistical difference, as shown in Table 5.

**Table 5:** General information of patients with stage 2 PI in both groups (*N* = 70).

Item	Pre-evidence application group [*n* (%)]	Post-evidence application group [*n* (%)]	*F/t*	*p*
Patient gender				
Male	18 (51.40)	23 (65.7)	2.087^1^	0.149
Female	17 (49.60)	12 (34.3)		
Age of patients (years)	59.49 ± 17.22	59.71 ± 13.96	2.162^2^	0.146
BMI				
lean	6 (17.14)	0(0.00)	−1.707^3^	0.088
Normal	19 (54.29)	18 (51.40)		
Overweight	10 (28.57)	17 (49.60)		
Education level				
Elementary school and below	12 (34.29)	7 (20.00)	−1.896^3^	0.058
Junior high school	7 (20.00)	5 (14.29)		
High School	10 (28.57)	10 (28.57)		
Junior college and college	3 (8.57)	9 (25.70)		
Undergraduate and above	3 (8.57)	4 (11.42)		
Whether smoking				
Yes	21 (60.00)	24 (68.57)	2.885^1^	0.069
No	14 (40.00)	11 (31.43)		
PI source				
within section	16 (45.71)	22 (62.86)	2.072^1^	0.150
brought in	19 (54.29)	13 (37.14)		
Underlying disease				
Yes	23 (65.71)	25 (71.40)	0.265^1^	0.607
None	12 (34.29)	20 (28.60)		
Wound location				
Sacrococcygeal	20 (57.14)	14 (40.00)	−1.059^3^	0.289
Hip	2 (5.71)	3 (8.57)		
Sciatic tubercle	4 (11.43)	2 (5.71)		
Other	9 (25.71)	16 (45.71)		
Braden score	11.86 ± 3.93	10.94 ± 3.56	0.249^2^	0.619
PUSH score	10.17 ± 2.70	11.0 ± 1.82	−1.559^2^	0.124
VAS score	8.91 ± 1.12	8.89 ± 0.96	0.114^2^	0.909

^1^Person chi-square test; ^2^two independent samples *t*-test; ^3^Mann–Whitney *U* rank sum test.

### Effect of evidence application at the patient level

#### Generalized estimating equation analysis of the level of PUSH score in the two groups of patients

The total PUSH score and the scores of each dimension at each time point before and after the intervention of the two groups of patients did not satisfy normal distribution (Shapiro–Wilk normality test), so it was not suitable to use repeated measures ANOVA, and instead, generalized estimating equations analysis of repeated measurements information was used. The test statistic is the value shown in Table 6. (i) Between-group effect: the total PUSH score and the area of PI of the two groups of patients and exudate volume as well as traumatic tissue type were statistically different (*p* < 0.05); that is, there was a significant difference in the effect and influence of the evidence before and after its application on the total PUSH score of patients’ PIs as well as the PI area, exudate volume, and traumatic tissue type; ① Within-group effect: there was a difference in the total PUSH score of the PIs of patients in the two groups and the scores of the dimensions in the two groups in each time point (*p* < 0.05); that is, there was a significant difference between the PUSH Total score and each dimension score measured at different time points differed significantly; (iii) Interaction effect: there was an interaction effect between the two groups of patients’ PUSH total score and PI area, exudate volume, and traumatic tissue type scores at time and between subgroups (*p* < 0.05), indicating that the two groups of patients’ PUSH total scores, PI area, exudate volume, and traumatic tissue type dimensions had different trends at different time points.

**Table 6:** Generalized estimation equation analysis of the level of PUSH scores in the two groups of patients (*N* = 70).

Item	Between-group effect		Within-group effect		Interaction effect	
	Wald *X*^2^	*p*	Wald *X*^2^	*p*	Wald *X*^2^	*p*
Total score	12.179	<0.001	330.050	<0.001	38.310	<0.001
PI area score	6.142	0.013	823.856	<0.001	103.282	<0.001
Exudate volume	20.577	<0.001	190.095	<0.001	19.940	<0.001
Wound tissue type	18.804	<0.001	129.764	<0.001	10.124	0.006

#### Intergroup comparison of PUSH scores at different time points between the two groups of patients

There was an interaction between the total PUSH scores and specific dimension scores of the two patient groups, and the differences in PUSH scores between the two groups at various time points were further analysed. The results showed that there was no statistically significant difference in the total PUSH score and the dimension scores between the two patient groups before the application of evidence (*p* > 0.05). The exudate volume dimension was lower than that of the control group at 1 week of wound treatment (*p* < 0.05); the total PUSH score and the scores of all dimensions except the exudate volume dimension were significantly lower than that of the control group after 2 weeks of wound treatment (*p* < 0.05), with a very large effect size for the total score difference at *T*₂ (Cohen’s *d* = 2.45, 95% CI [1.66, 3.23]), as shown in Table 7.

**Table 7:** Intergroup comparison of PUSH scores at different time points between the two groups (*N* = 70).

Item	Time	Group (I)	Group (J)	Mean difference (I-J)	Standard error	*p*	95% confidence interval	
							Lower limit	Upper limit
Total score	*T*_0_	Control group	Intervention group	1.183	0.546	0.075	0.113	2.252
	*T*_1_	Control group	Intervention group	0.457	0.664	0.493	−0.867	1.781
	*T*_2_	Control group	Intervention group	4.147	0.622	<0.001	2.904	5.390
Area	*T*_0_	Control group	Intervention group	0.429	0.480	0.375	−1.386	0.529
Area	*T*_1_	Control group	Intervention group	0.714	0.578	0.221	−0.439	1.868
	*T*_2_	Control group	Intervention group	3.265	0.515	<0.001	2.236	4.294
	*T*_0_	Control group	Intervention group	0.657	0.134	0.666	−0.928	−0.387
Exudate volume	*T*_1_	Control group	Intervention group	0.457	0.140	0.001	−0.732	−0.182
	*T*_2_	Control group	Intervention group	0.118	0.102	0.248	−0.082	0.317
Wound tissue type	*T*_0_	Control group	Intervention group	0.114	0.187	0.543	−0.259	0.487
	*T*_1_	Control group	Intervention group	0.257	0.148	0.087	−0.038	0.553
	*T*_2_	Control group	Intervention group	0.765	0.115	<0.001	0.535	0.994

*T*_0_, *T*_1_, and *T*_2_ denote at the time of first wound treatment, at 1 week of wound treatment, and at 2 weeks of wound treatment, respectively.

#### Generalized estimating equation analysis of VAS score and wound healing rate at different time points in the two groups of patients

The VAS scores and wound healing rates of the two groups of patients at various time points before and after the intervention did not satisfy normal distribution, so it was not suitable to use repeated measures ANOVA, but to use generalized estimating equations analysis of repeated measures information, and the test statistic was the Wald^2^value, as shown in Tables 8, 9. Between-group effect: there was a statistically significant difference in the VAS scores and wound healing rates of the two groups of patients (*p* < 0.05); that is, the use of the there was a statistically significant difference between the two groups in VAS score and wound healing rate (*p* < 0.05), i.e., there was a significant difference in the effect and influence on patients’ VAS and wound healing rate before and after the adoption of the action strategy. There was an interaction effect (*p* < 0.05) indicating that the two groups of patients had different trends in terms of VAS score and PI wound healing rate at different time points. At *T*₂ (2 weeks of wound treatment), the VAS score in the intervention group (0.68 ± 0.81) was significantly lower than that in the control group (3.35 ± 2.42), with a large effect size (Cohen’s *d* = 1.52, 95% CI [0.95, 2.08]).

**Table 8:** Generalized estimating equation analysis of VAS scores at different time points for both groups (*N* = 70).

Group	*T*_0_	*T*_1_	*T*_2_	Wald *X*^2^ (between groups)	Wald *X*^2^ (within group)	Wald *X*^2^ (interaction)
Control group	8.91 ± 1.12	6.06 ± 2.31	3.35 ± 2.42	29.979	679.944	27.347
Intervention group	9.00 ± 0.69	5.09 ± 1.25	0.68 ± 0.81	<0.001	<0.001	<0.001
*t*-value	−1.809	2.188	6.111			
*p*-value	0.075	0.032	<0.001			

*T*_0_, *T*_1_, and *T*_2_ denote at the time of first wound treatment, at 1 week of wound treatment, and at 2 weeks of wound treatment, respectively.

**Table 9:** Generalized estimation equation analysis of wound healing rate at different time points in both groups (*N* = 70).

Group	*T*_0_	*T*_1_	*T*_2_	Wald *X*^2^ (between groups)	Wald *X*^2^ (within group)	Wald *X*^2^ (interaction)
Control group	–	40.23 ± 28.29	76.01 ± 23.33	26.579	768.64	13.366
Intervention group	–	60.06 ± 20.61	95.33 ± 17.14	<0.001	<0.001	0.001
*t*	–	−3.352	−3.891			
*p*	–	0.001	<0.001			

*T*_0_, *T*_1_, and *T*_2_ indicate the time of first wound treatment, 1 week of wound treatment, and 2 weeks of wound treatment, respectively.

#### Individual variation in treatment response and adverse events

To further evaluate the robustness and safety of the intervention protocol, this study monitored individual patient healing outcomes and adverse events. Wound healing rates <50% at 2 weeks post-intervention were defined as “delayed healing.” Results are shown in Table 10: no delayed healing or adverse events occurred in the intervention group (*n* = 35). In contrast, three patients (8.6%) in the control group (*n* = 35) experienced delayed wound healing. The absolute risk reduction for delayed healing was 8.6% (95% CI [0.2, 18.5%]).

**Table 10:** Comparison of individual patient healing outcomes and adverse events.

Group	*N*	Number of delayed healing cases *n* (%)	Number of adverse event cases *n* (%)	Remarks
Control group	35	3 (8.6%)	1 (2.9%)	Mild skin erythema, relieved after dressing change
Intervention group	35	0 (0.0%)	0 (0.0%)	–

## Discussion

Pressure ulcers are a common complication among long-term bedridden hospitalized patients, significantly impacting patients’ physical, psychological, economic, and quality of life. They also profoundly affect the efficiency of acute care services and the healthcare system (11, 41–43). The primary objective of this study was to address inconsistencies in international guidelines for stage 2 PI management, identify barriers to clinical translation, and address localization issues that fail to align with China’s healthcare context. While existing guidelines provide a principled framework, they lack operationalized, unified protocols for standardized wound assessment processes, systematic implementation of pain management, and particularly for cost-effective selection strategies that integrate China’s medical insurance catalog with commonly used hospital dressings. This deficiency results in low compliance among non-wound specialty nurses and significant variations in clinical practice. This study systematically refined and localized evidence to align and elaborate on international guidelines in the following critical areas: (1) For wound assessment, standardized tools such as PUSH and DESIGN were mandated alongside standardized measurement protocols, consistent with recommendations from the WHS and the NPIAP (44). Additionally, QR code dressing manuals and optimized electronic health record (EHR) data entry modules addressed the “implementation adherence” challenge not covered in the guidelines; (2) For pain management, “assessing pain before and after each dressing change” is established as a review indicator. Non-pharmacological interventions are integrated, and assessment items are embedded into the electronic medical record system, transforming guideline principles into standardized, clinically mandatory steps; (3) For dressing selection, we synthesized international evidence-based research while closely aligning with China’s medical insurance reimbursement policies and hospital dressing catalogs (1, 25, 26). This established clear, cost-effective, and feasible selection pathways based on wound exudate levels, achieving a substantive translation from high-level evidence to locally applicable cost-effective practice. Furthermore, the refinement and widespread adoption of these measures will effectively reduce hospital stays, enhance the quality of acute care services, alleviate pressure on the healthcare system, and conserve medical resources (41–43). Future multi-center validation studies can further evaluate the clinical benefits of these interventions while exploring the integration of digital technologies to promote patient self-management and improve the efficiency of guideline implementation.

In this phase, by comparing the wound exudate, area, tissue type and wound healing rate of hospitalized patients with PI in 2 phases before and after the evidence-based practice, the results showed that the total PUSH score of patients with PI was significantly reduced after the evidence was applied at the first wound treatment, at the time of 1 week of wound treatment, and at the time of 2 weeks. The wound healing rate of patients with PI was significantly improved compared with that before the evidence was applied. The difference was statistically significant, which indicated that the action strategy was effective. Tang Yifan and other studies have also shown that PI evidence-based nursing practice programs can promote wound healing in patients (15, 31, 45). Previously published guidelines have described wound management inconsistently; this study provides a precise description of stage 2 PI wounds assessment, the choice of tissue cleansing methods, and wound documentation, thereby establishing a basis for standardized, high-quality care of stage 2 PI wounds. It is essential to recognize that several factors influence the ability to heal wounds and vary from person to person. In addition, the Australian Wound Organization suggests that effective wound healing can be demonstrated if a quantitative indicator of a 20–40% reduction in wound area is achieved within 1 month (46). Therefore, this study emphasized the importance of timely documentation when assessing wounds for dynamic wound monitoring.

Studies have shown that poor compliance with pain assessment increases uncertainty during wound management, leading to difficult pain diagnosis and management decisions, ultimately rendering pain relief ineffective and increasing patient harm (47). The review of the current situation revealed that only 15% of the wound liaisons performed pain evaluations at each wound management. There were no skin liaisons who took appropriate therapeutic measures based on the results of the pain evaluation, which is consistent with the results of the study conducted by Zhang (48). Analysis indicates that the primary causes are related to the lack of a systematic assessment process and the increased workload associated with assessments. To address these issues, a combined strategy at both the system and practice levels is recommended: At the system level, integrate structured pain assessment components into the hospital HIS system, enabling real-time assessment and documentation via handheld PDA barcode scanning. At the practice level, nurses are advised to follow a structured micro-process of “Ask-Check-Record-Act” during each dressing change: Proactively inquire about and quantify pain before dressing changes; observe nonverbal pain signs during procedures; immediately record scores and characteristics; and take action based on results—such as using music for distraction, selecting pain-free dressings like silicone-backed or foam options, or employing pain-relief techniques like parallel removal after saline soaking. For moderate to severe refractory pain, nurses should promptly initiate escalation and collaboration protocols. This integrated strategy combines systemic support with standardized practices, ensuring evidence-based solutions translate into actionable bedside interventions (49, 50).

This study’s in-depth analysis of individual efficacy and safety reveals the added value of evidence-based action strategies in managing complex cases. The intervention group reported zero instances of delayed healing or adverse events, whereas the control group experienced three cases (8.6%) of delayed healing, one of which progressed to confirmed wound infection. This stark contrast not only confirms the overall efficacy of the protocol but also underscores its robustness in ensuring consistent outcomes and preventing complications. Retrospective analysis revealed that all three delayed healing cases in the control group were associated with poorly controlled type 2 diabetes, suggesting that systemic factors combined with inadequate local wound care constitute a high-risk profile for delayed healing (51). Thus, the value of this protocol extends beyond providing clear operational guidelines for non-specialist nurses. It functions as a structured clinical decision support system, empowering them to identify and manage additional risks associated with comorbidities, such as diabetes. This approach provides an additional layer of safety for all patients—particularly those with complex cases and high-risk factors—effectively preventing their wounds from progressing to chronic, difficult-to-heal conditions.

Studies have shown that clinical nurses’ knowledge of wounds is most often derived from previous work experience, which often results in inadequate updating of wound-related knowledge or incomplete wound assessment content (52). The results of this study showed that after conducting evidence-based practice, the nurses’ knowledge of PI questionnaire scores improved from (53.31 ± 4.75) to (56.29 ± 4.72), with a statistically significant difference (*p* < 0.05), suggesting that the use of best evidence has led to an effective improvement in nursing staff’s knowledge of PI wound management. This finding is consistent with previous research demonstrating that educational interventions for nurses can significantly enhance PI-related knowledge, which in turn leads to improved clinical outcomes. For instance, one study reported a statistically significant decrease in the overall prevalence of PI (28.7%) following an educational intervention, highlighting the success of such initiatives and underscoring the importance of educating nurses on PI practices. These improvements are likely associated with enhanced nurse awareness resulting from tailored, evidence-based educational interventions (12). In this study, by carrying out online and offline training, filming popular science videos, and producing an electronic version of the wound dressing manual, nursing care was made more refined so that clinical nursing staff gained sufficient theoretical and practical knowledge of PI, which led to a significant improvement in their knowledge, and thus a significant increase in the rate of reviewing the implementation of indicators. Following one round of review, the level of PI knowledge among nursing staff was significantly improved, thereby enhancing patient health outcomes. In addition, it has been suggested that focus should be placed on the importance of patients’ and caregivers’ health literacy and self-efficacy in clinical practice for program implementation (53, 54).

Therefore, in future studies, we can narrow the cognitive gap between physicians and patients by strengthening the level of health knowledge and self-efficacy of patients and caregivers, which will ultimately promote the implementation of the program and improve the clinical symptoms of patients In the future, targeted health education on wound care practices can be conducted for physicians and patients. Health education tools, such as information technology, experiential education, and positive thinking interventions, can be enhanced to promote their use and fully leverage the clinical application of PI management best practices (55–57).

The results of this study showed a significant increase (*p* < 0.05) in the implementation rate of all 15 review indicators after the application of the evidence, with three of them increasing from 0 to 100%. The evidence-based practice of phase 2 PI wound management is a continuous quality improvement process, and in this study, through homogenization training, system optimization, production of wound record forms and training assessment, nurses “internalized in their minds and externalized in their actions,” which improved the training effect and ensured the implementation rate of the review indicators after the application of evidence. The results show that indicators 7, 8, and 9 are not implemented. The results show that the implementation rates of indicators 7, 8, 9, 10, and 18 are still lower than 80%, although there has been a significant increase. Analyzing the reasons, first, it may be related to the prolonged working hours during pain assessment and non-pharmacological interventions, which leads to a decrease in the efficiency of healthcare workers; second, it may be related to the fact that nursing staff are still in the stage of familiarity with the process of wound treatment in PI. It is recommended that clinical managers encourage caregivers more and increase the perception of the importance of pain management for PI wound healing, thus increasing compliance with pain management and motivation for treatment.

In this study, we developed and improved the relevant contents for the system level, mainly including the optimization of the system, the wound record form, the wound dressing manual, quality management, training and assessment of the best evidence in five aspects, and reformed the action strategy of the inpatient stage 2 PI wound management through the way of evidence-based practice, from the improvement of the quality control mechanism, process reengineering, carrying out the relevant training, and the improvement of the hardware facility innovations, which resulted in enhanced review of PI implementation rates, improved patient wound regression, and ultimately improved clinical outcomes for patients. From the baseline survey, it was concluded that clinical nurses in our hospital had not yet paid enough attention to the wound management of patients with stage 2 PI, and the department lacked relevant wound/pain-related assessment tools, manuals for wound cleansing and the use of relevant dressings, and the system of PI reporting was labour-intensive; therefore, we worked with the Information Department to improve the PI reporting and management system, and to form a wound record sheet in order to understand the important changes, evaluate the progress of healing, and adjust the therapeutic Decision-making. At the operational level, we provided nursing staff with supporting equipment, including operating procedures, inspection standards, and measurement tools, which improved the standardisation of nursing staff operations and enabled them to implement PI wound management measures better.

### Limitations

Although comprehensive guidance documents, such as the 2023 WHS guidelines, exist internationally, their successful application depends on a deep integration with local clinical settings and resources (44). This study constructed a 2-phase PI chain management model led by non-specialized nurses, which effectively improved the quality of care through evidence-based practice. However, this study still has several limitations. First, regarding sampling methods, although we included all skin liaisons from the pilot units to maximize internal feasibility and avoid selection bias, the single-center design and non-probability sampling approach necessitate caution when generalizing findings to other hospitals or nursing populations with different backgrounds. Second, although no significant differences were observed between the two patient groups in key baseline characteristics, such as comorbidities and BMI, which enhanced comparability between groups, this study did not actively control for or randomly assign potential confounding factors, including comorbidities, specific medications, or more detailed nutritional indicators. This may have influenced the results. Additionally, the development of specialized nursing in China itself faces challenges such as insufficient training for non-specialized personnel and weak professional awareness, which have impacted the depth and sustainability of evidence translation to some extent. Meanwhile, the COVID-19 pandemic has constrained the sample size and duration of studies; future research requires larger-scale, long-term follow-ups to validate the stability of outcomes. Finally, exploring novel nursing models such as intelligent monitoring and remote guidance represents a key direction for future research (58). The study confirms that systematic training, process standardization and information support can significantly enhance the execution of norms by nursing staff and improve patient prognosis. Follow-up should focus on strengthening pain management compliance, optimizing nursing resource allocation, and establishing a long-term quality improvement mechanism to provide sustainable solutions for managing stress injuries.

## Conclusion

This study developed an action strategy for stage 2 pressure ulcer wound management based on best evidence and validated its scientific rigor and feasibility through evidence-based practice. Results demonstrated that implementing this strategy significantly enhanced nurses’ pressure ulcer knowledge, as indicated by a PZ-PUKT score increase from 53.31 ± 4.75 to 56.29 ± 4.72 (*p* < 0.01). Additionally, the implementation rate of review indicators increased from 0 to 56.25% at baseline to 70–100%. Patients’ wound PUSH total scores, affected area, exudate volume, and tissue type scores all showed significant improvement (*p* < 0.05), with enhanced wound healing rates and reduced pain scores. These findings indicate that systematic training, standardized processes, and information support can effectively enhance wound management capabilities among non-wound specialty nurses and optimize clinical practice. Limitations of this study include a small sample size and limited training resources. Future efforts should focus on strengthening pain management compliance and exploring new nursing models, such as intelligent monitoring, to establish a long-term quality improvement mechanism. Overall, this strategy provides evidence-based support for standardized management of stage 2 PI and contributes to improved patient health outcomes.

## Data Availability

The original contributions presented in the study are included in the article/Supplementary material, further inquiries can be directed to the corresponding authors.
