# Supplementary material for: Evidence-based management of stage 2 pressure injuries in country-specific context
Source: Front Med (Lausanne). 2026 Jan 12;12:1650052. doi: 10.3389/fmed.2025.1650052 (PMC12832647; doi:10.3389/fmed.2025.1650052)
Supplement: Supplementary file 1 [file Table_1.docx]

Complete the initial skin assessment within one hour of admission.

Is there any pressure injury?

Determine the stage of the injury

Implement routine skin
 care and prevention measures

Is it Stage 1/2?

Initiate standardized wound management protocol

Assessment: Color, Temperature, Integrity, Measurement, Pain

Cleaning: Use mild cleanser, gently rinse

Treatment: Select dressing based on exudate/infection status

Documentation: Complete standardized documentation form and take photographs

Enter the ongoing monitoring and follow-up process

Wound Healing

The department's skin
 coordinator

Under the guidance of the department's dermatology
 Protection

Quality Assurance and Support System

System Optimization: Information-based Evaluation
 and Reporting System

Training Support: Online and Offline
 Integrated with Operational Videos

Tool Support: Standardized Record Forms
 and dressing manuals

Mechanism safeguards: Regular reviews,
 incentives, and online Q&A

No, ≥3 phases

No

Yes

Apply Now for In-Hospital Wound
 Therapist Consultation

Implement specialized treatment plan
